# Vertical transmission of highly similar *bla*_CTX-M-1_-harboring IncI1 plasmids in *Escherichia coli* with different MLST types in the poultry production pyramid

**DOI:** 10.3389/fmicb.2014.00519

**Published:** 2014-09-30

**Authors:** Katrin Zurfluh, Juan Wang, Jochen Klumpp, Magdalena Nüesch-Inderbinen, Séamus Fanning, Roger Stephan

**Affiliations:** ^1^Institute for Food Safety and Hygiene, Vetsuisse Faculty, University of ZurichZürich, Switzerland; ^2^UCD Centre for Food Safety, School of Public Health, Physiotherapy and Population Science, UCD Centre for Molecular Innovation and Drug Discovery, University College DublinDublin, Ireland; ^3^Institute of Food, Nutrition and Health, Swiss Federal Institute of Technology in ZürichZürich, Switzerland

**Keywords:** *E. coli*, plasmid sequencing, CTX-M-1, poultry production pyramid, IncI1, conjugation

## Abstract

**Objectives**: The purpose of this study was to characterize sets of extended-spectrum β-lactamases (ESBL)-producing *Enterobacteriaceae* collected longitudinally from different flocks of broiler breeders, meconium of 1-day-old broilers from theses breeder flocks, as well as from these broiler flocks before slaughter.

**Methods**: Five sets of ESBL-producing *Escherichia coli* were studied by multi-locus sequence typing (MLST), phylogenetic grouping, PCR-based replicon typing and resistance profiling. The *bla*_CTX-M-1_-harboring plasmids of one set (pHV295.1, pHV114.1, and pHV292.1) were fully sequenced and subjected to comparative analysis.

**Results**: Eleven different MLST sequence types (ST) were identified with ST1056 the predominant one, isolated in all five sets either on the broiler breeder or meconium level. Plasmid sequencing revealed that *bla*_CTX-M-1_ was carried by highly similar IncI1/ST3 plasmids that were 105 076 bp, 110 997 bp, and 117 269 bp in size, respectively.

**Conclusions**: The fact that genetically similar IncI1/ST3 plasmids were found in ESBL-producing *E. coli* of different MLST types isolated at the different levels in the broiler production pyramid provides strong evidence for a vertical transmission of these plasmids from a common source (nucleus poultry flocks).

## INTRODUCTION

One of the currently most important antibiotic resistance mechanisms in *Enterobacteriaceae* is based on plasmid-mediated production of extended-spectrum β-lactamases (ESBLs) which inactivate β-lactam-antibiotics including cephalosporins and monobactams by hydrolyzing their β-lactam ring (Frère et al., 1992). ESBLs are classified according to their primary sequences and substrate profiles into different families such as the TEM-, the SHV-, the OXA-, and the CTX-M -family (Bush and Jacoby, 2010). Currently, CTX-M enzymes are the most widespread ESBLs (Naseer and Sundsfjord, 2011). Of the more than 150 different CTX-M sequence types (ST) that are known and filed in the Lahey database ^1^, CTX-M-1 predominates in *Escherichia coli* isolated from food-producing animals and foods in Europe (EFSA, 2011).

Recently, several reports highlighted the dissemination of ESBL-producing *E. coli* in poultry in several countries across Europe (Smet et al., 2008; Cortés et al., 2010; Randall et al., 2011; Wasyl et al., 2012) or in chicken meat (Leverstein-van Hall et al., 2011; Overdevest et al., 2011; Kola et al., 2012). A high fecal prevalence of *bla*_CTX-M-1_-harboring *E. coli* has also been reported for poultry flocks in Switzerland (Endimiani et al., 2012; Geser et al., 2012). Moreover, CTX-M-1-producing *E.* coli have also been found both on local and imported retail poultry meat (Abgottspon et al., 2014; Vogt et al., 2014).

The high occurrence of ESBL-producing bacteria in Swiss poultry flocks, however, cannot be explained by the use of high amounts of antimicrobials for broilers, since on average only 1 of 10 poultry flocks need to be treated with antibiotics and when treatment is indicated, fluoroquinolones and not β-lactam-antibiotics are the drugs of choice (Büttner et al., 2013).

As hypothesized by others (Dierikx et al., 2013; Agersø et al., 2014; Nilsson et al., 2014), one possible explanation for this situation could be the existence of a common source (nucleus poultry flocks), disseminating ESBL-producers vertically along the poultry chain over large geographical regions. ESBL-producing *Enterobacteriaceae* might be imported by one-day-old broiler breeders and then spread from the top to the bottom of the production pyramid. We hypothesize that a specific *E. coli* type is unlikely to be responsible for this occurrence, but that a limited number of different plasmids transferred between different *E. coli* types by conjugation are involved.

The purpose of this study was to characterize sets of ESBL-producing *Enterobacteriaceae* collected longitudinally from different flocks of broiler breeders, meconium of one-day-old broilers from theses breeder flocks, as well as from these broiler flocks before slaughter, and to sequence plasmids harboring the *bla*_ESBL_ genes from one set of isolates.

## MATERIALS AND METHODS

### BACTERIAL ISOLATES

From July 2013 through May 2014 boot socks (gauze socks walked over the length of a broiler chicken house) of parental broiler breeder flocks (from grandparent breeders from France), meconium of 1-day-old broilers from these breeder flocks and boot socks of these broilers flocks taken before slaughter were sampled longitudinally through the production pyramid. Boot socks and the meconium were enriched for 24 h at 37°C in 250 and 10 ml of EE Broth (BD, Franklin Lakes, NJ, USA), respectively. Thereafter, one loopful each of the enrichment cultures was inoculated onto chromogenic Brilliance ESBL agar (Oxoid, Hampshire, UK) and incubated at 37°C for 24 h under aerobic conditions. All colonies with different color and morphology were picked from the selective plates and sub-cultured on triple sugar iron-agar (TSI) agar (Oxoid) at 37°C for 24 h. Oxidase-negative isolates were subjected to identification by API ID 32 E (bioMérieux, Marcy l’Etoile, France). One isolate per sample was selected for further characterization.

### ANTIBIOTIC SUSCEPTIBILITY TESTING AND PHENOTYPIC ESBL DETECTION

Susceptibility testing was performed by agar diffusion methods, using antibiotic disks (Becton Dickinson and Company, Maryland, USA) and ESBL ellipsometer-test (E-test) strips (bioMérieux, Marcy l’Etoile, France), according to the manufacturers’ protocols. Results were interpreted according to the criteria of the Clinical and Laboratory Standards Institute (CLSI, 2011).

### IDENTIFICATION OF *bla*_ESBL_ GENES

Bacterial strains confirmed as producing ESBLs were further analyzed by PCR. DNA was purified using a standard heat lysis protocol (Sambrook and Russel, 2006). Thereafter, five specific primer sets (custom-synthesized by Microsynth, Balgach, Switzerland) were used to screen for β-lactamase-encoding genes belonging to the *bla*_TEM_, *bla*_SHV_, and *bla*_CTX-M_ families (Pitout et al., 1998; Geser et al., 2012).

Resulting amplicons were purified using the GenElute^TM^ PCR Clean-Up (Sigma–Aldrich, Buchs, Switzerland) according to the manufacturer’s recommendations. Custom-sequencing was performed by Microsynth (Balgach, Switzerland) and the nucleotide- and translated protein-sequences were analyzed with CLC Main Workbench 7.0.2 (CLC bio, Aarhus, Denmark). For database searches the BLASTN program of NCBI ^2^ was used.

### MLST OF ESBL PRODUCERS

Internal fragments of seven housekeeping genes (*adk, fumC, gyrB, icd, mdh, purA, and recA*) were sequenced (Wirth et al., 2006) and allele and ST were assigned in accordance with the *E. coli* multi-locus sequence typing (MLST) website ^3^.

### DETERMINATION OF *E. coli* PHYLOGENETIC GROUPS

*Escherichia coli* isolates were subjected to phylogenetic grouping by PCR as described previously (Clermont et al., 2000). Thereby, *E. coli* strains are assigned to four main phylogenetic groups (A, B1, B2, and D). Groups A and B1 contain commensal isolates and members of the group B2 and D are classified as virulent extra-intestinal strains which often carry pathogenicity associated genes.

### PCR-BASED REPLICON TYPING

Plasmid incompatibility (Inc) types were determined by PBRT (Carattoli et al., 2005; Villa et al., 2010), using genomic DNA as template.

### CONJUGATION MATING EXPERIMENTS

Conjugation experiments were performed with the plasmid-free recipient strain *E. coli* HK225 (Strep^r^, Rif^r^; Kayser et al., 1982). Briefly, single colonies of the donor and recipient were inoculated in LB broth (Difco Laboratories) and grown overnight at 37°C. Subsequently, equal volumes of the donor and recipient cultures were mixed and incubated overnight at 37°C without shaking. Serial dilutions were then plated on LB agar (Difco Laboratories) selection plates supplemented with a combination of 600 μg/ml streptomycin (Sigma–Aldrich, Buchs, Switzerland) 100 μg/ml rifampicin (Sigma–Aldrich) and 10 μg/ml cefotaxime (Sigma–Aldrich).

The conjugation frequency per donor was determined by plating serial dilutions of the mating on selective plates on which the donor strain and the transconjugant can grow (LB-agar supplemented with 10 μg/ml cefotaxime) as well as on plates on which only the transconjugants can grow (LB-agar supplemented with 600 μg/ml streptomycin, 100 μg/ml rifampicin, and 10 μg/ml cefotaxime). The transfer frequency was calculated as the quotient of the number of transconjugants over the number of transconjugants plus donors.

### PLASMID EXTRACTION AND SEQUENCING

The plasmids pHV295.1, pHV114.1, and pHV292.1 were purified using the Qiagen Large-Construct Kit (Qiagen, Hombrechtikon, Switzerland) according the manufacturer’s protocol. The complete plasmid sequence was determined using the Pacific Biosciences SMRT sequencing approach (Functional Genomics Center, Zurich, Switzerland) applying P4/C2 chemistry on an RSII device. A 120 min movie was recorded from a 10 kb insert library per cell.

### BIOINFORMATICS

Sequence assembly was carried out using the SMRT-Analysis software version 2.0 with the RS_HGAP_Assembly.1 algorithm and default settings. The plasmid sequence was automatically annotated using the online Rapid Annotation Subsequencing Technology (RAST; Aziz et al., 2008) and CLC Main Workbench Version 7.0.2 (CLC bio, Aarhus, Denmark). Automated annotation was manually refined.

### NUCLEOTIDE SEQUENCE

The GenBank accession numbers for pHV295.1, pHV114.1, and pHV292.1 plasmids are KM377238, KM377239, and KM377240, respectively.

## RESULTS

### PHENOTYPIC AND GENOTYPIC CHARACTERISTICS OF THE ESBL-PRODUCING ISOLATES

Five sets of ESBL-producing *Enterobacteriaceae* collected longitudinally from five different flocks of broiler breeders, meconium of 1-day-old broilers from these breeder flocks as well as from these broiler flocks before slaughter were further characterized. Species identification revealed that all were *E. coli* and all harbored *bla*_CTX-M-1_ genes (**Table 1**). Phylogenetic grouping and MLST analysis showed that different *E. coli* types could be identified at the investigated production levels in all five sets, suggestive of a non-clonal bacterial composition. *E. coli* B1 ST1056 was isolated in all five sets either in the broiler breeder samples or in the meconium samples. Eleven of the 15 isolates belonged to commensal phylogenetic groups A and B1; only four isolates belonged to the virulent extra-intestinal *E. coli* group B2 and D. In total, 11 different MLST ST were detected (**Table 1**).

**Table 1:** Phenotypic and genotypic characteristics of the extended-spectrum β-lactamases (ESBL)-producing isolates from flocks of broiler breeder, meconium of 1-day-old broilers and broilers.

	Sample	Production level	Species	Phylogenetic group	MLST ST	Disc diffusion test [mm]														Plasmid incompatibility types
						AM	AMC	CF	CTX	GM	K	S	TE	CIP	NA	C	SMZ	TMP	
SET 1	HV295.1	Broiler breeders	*E. coli*	B1	ST 109	**6**	20	**6**	16	22	24	18	21	30	28	28	**6**	**6**		I1, F
	HV114.1	Meconium	*E. coli*	B1	ST 1056	**6**	19	**6**	18	25	25	13	**6**	29	26	27	**6**	**6**		I1, F
	HV292.1	Broiler	*E. coli*	B1	ST 602	**6**	18	**6**	17	20	22	12	**6**	30	28	22	**6**	**6**		I1, F
SET 2	HV183	Broiler breeders	*E. coli*	B1	ST 1056	**6**	16	**6**	15	22	21	15	23	>30	25	26	**6**	**6**		I1, F
	HV226	Meconium	*E. coli*	B1	ST 1146	**6**	18	**6**	15	22	26	16	24	30	25	25	**6**	**6**		I1, F
	HV297.1	Broiler	*E. coli*	A	ST 10	**6**	19	**6**	16	22	24	17	22	>30	28	25	**6**	**6**		I1, F
	HV84.1	Meconium	*E. coli*	B1	ST 1056	**6**	18	**6**	15	23	24	12	**6**	>30	26	26	**6**	**6**		I1, F
	HV290.1	Broiler	*E. coli*	D	ST 117	**6**	21	**6**	18	22	24	17	23	>30	28	24	**6**	**6**		I1. F
SET 4	HV337.1	Broiler breeders	*E. coli*	B1	ST 1056	**6**	17	**6**	15	21	21	16	21	30	26	25	**6**	**6**		I1, F
	HV359.1	Meconium	*E. coli*	B2	ST 355	**6**	17	**6**	15	23	22	16	23	28	**6**	24	**6**	**6**		I1, F
	HV403.1	Broiler	*E. coli*	A	ST 1112	**6**	19	**6**	17	22	23	18	25	>30	28	24	**6**	**6**		I1, F
								**6**												
SET 5	HV338.1	Broiler breeders	*E. coli*	D	ST 1629	**6**	17		16	24	24	19	**6**	>30	28	29	**6**	**6**		I1, F
	HV369.1	Meconium	*E. coli*	B1	ST 1056	**6**	19	**6**	15	24	24	12	**6**	>30	26	27	**6**	**6**		I1, F
	HV420.1	Broiler	*E. coli*	A	ST 752	**6**	17	**6**	18	24	25	15	23	30	**13**	22	**6**	**6**		I1, F

*Resistances to specific antibiotics are in bold type. AM, ampicillin; AMC, amoxicillin-clavulanic acid; CF, cephalothin; CTX, cefotaxime; GM, gentamicin; K, kanamycin; S, streptomycin; TE, tetracycline; CIP, ciprofloxacin; NA, nalidixic acid; C, chloramphenicol; SMZ, sulfamethoxazole; TMP, trimethoprim; CT/CTL, cefotaxime/cefotaxime clavulanic acid; TZ/TZL, ceftazidime/ceftazidime clavulanic acid; PM/PML, cefepime/cefepime clavulanic acid. Multi-locus sequence typing (MLST) sequence type (ST).*

Phenotypic antibiotic resistance characterization indicated that all isolates were resistant to sulfamethoxazole and trimpethoprim in addition to the β-lactam antibiotics tested. Six isolates (two from the broiler breeders, three from meconium, one from a broiler) and two isolates (one each cultured from meconium and a broiler) were additionally resistant to tetracycline or nalidixic acid, respectively (**Table 1**).

PCR-based replicon typing showed that all isolates from the five sets possessed at least two plasmids: an IncI1 and an IncF replicon type plasmid. One isolate from broiler breeders (HV300) also carried an IncK plasmid (**Table 1**).

### CONJUGATION EXPERIMENTS, PLASMID SEQUENCING, AND COMPARATIVE ANALYSIS

**FIGURE 1:**
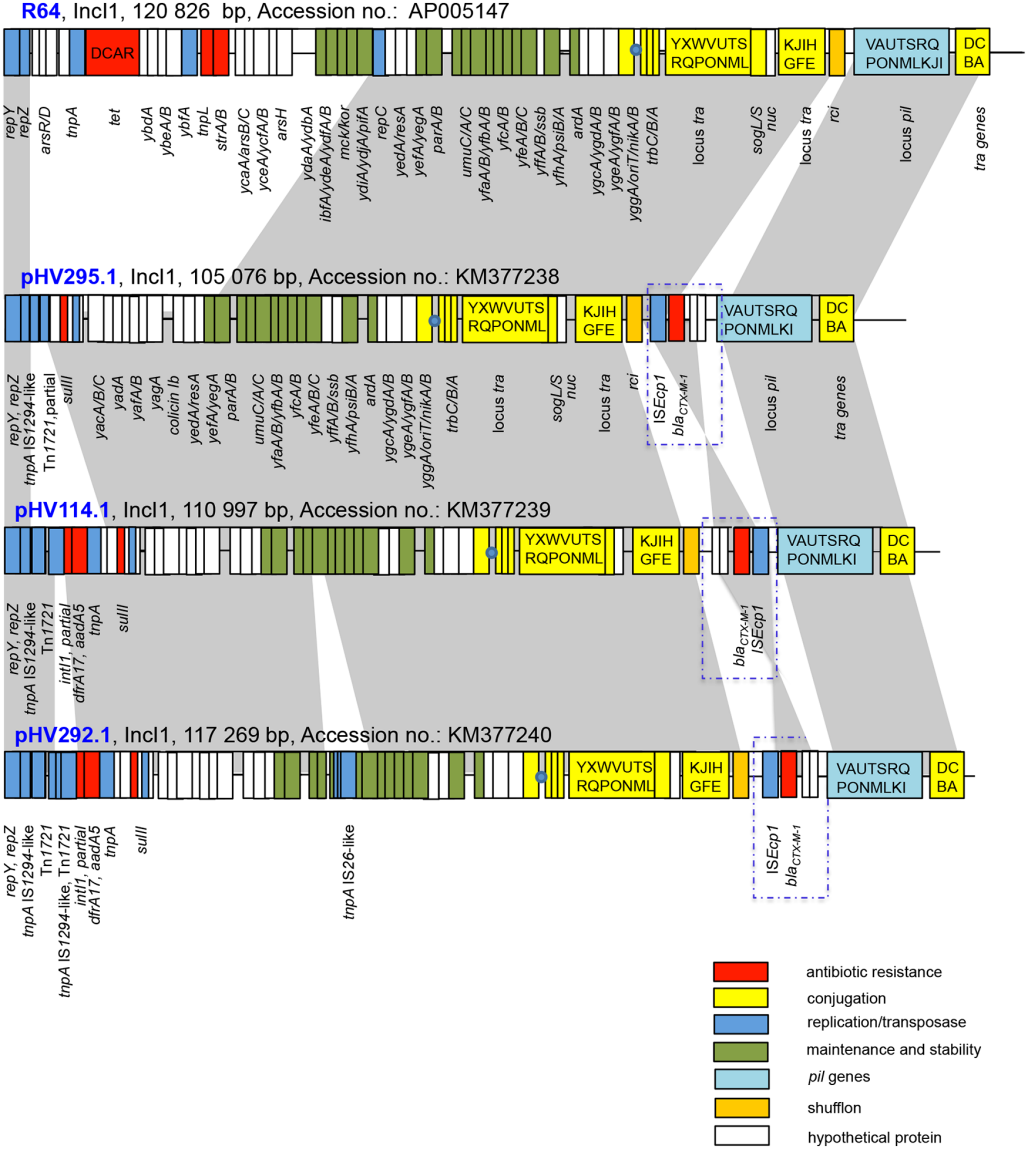
**Comparative analysis of pHV295.1, pHV114.1, and pHV292.1.** Major structural features of the IncI1 plasmids are shown in comparison with the IncI1 reference plasmid R64 (Accession no. AP005147). Gray shaded areas indicate homologies in the plasmid scaffold regions. Red boxes mark antibiotic resistance genes. Conjugation-related genes are shown with capital letters in yellow boxes. Blue boxes indicate transposon-, integron-, or replication associated genes. Green boxes denote maintenance- and stability-related genes. White boxes indicate hypothetical proteins and light blue boxes show *pil* genes. The origin of transfer *oriT* is shown as a blue circle. The figure is not drawn to scale.

Conjugation experiments were performed with the isolates from set 1 (comprising isolates HV295.1, HV114.1, and HV292.1). The *bla*_CTX-M-1_-carrying plasmids could be transferred to the recipient strain *E. coli* HK225 with conjugation frequencies of 1.35 × 10^-3^, 4.63 × 10^-3^, and 1.29 × 10^-5^ per donor cell, respectively. Whole plasmid sequencing revealed that all three plasmids belong to the incompatibility group IncI1 of plasmid pMLST ST3 type (García-Fernández et al., 2008). Plasmids pHV295.1, pHV114.1, and pHV292.1 were 105 076, 110 997, and 117 269 bp in size with overall G+C contents of 50.5, 51 and 51.3%, respectively. All three plasmids presented a similar backbone structure (nucleotide similarity 99%) that encoded for initiation of plasmid replication (*oriT* operon), conjugative transfer (*traABCD*), plasmid maintenance and stability (e.g., *yefA, parAB, impABC, ssb*,) and *pil* operon (Sampei et al., 2010). The latter encodes for a group of proteins forming a type IV pilus involved in the formation of donor-recipient cell aggregates (Kim and Komano, 1997). All of these modules were typical of those identified previously in IncI1 types and were also found on the IncI1 reference plasmid R64 (GenBank accession number AP005147.1; **Figure 1**).

Comparative analysis of the three plasmids sequenced in this study showed that they were very similar over the complete sequence length (**Figure 1**). Two regions are of main interest: (i) the region proximal of *repZ* (pHV295.1: 8.2 kb; pHV114.1: 14.1 kb, and pHV292.1: 17.6 kb) and (ii) a 2.7 kb module containing IS*Ecp1*-*bla*_CTX-M-1_ encoding the resistance to broad-spectrum cephalosporins inserted between *rci* and *pilVA*.

The region between *repZ* and *yacA* is the most variable region in the three plasmids and it consists of a number of mobile elements such as (partial) transposases, (partial) integrons and IS-elements. The plasmid isolated on the level of broiler breeders (pHV295.1) carried in this region a *sul2* gene that encodes for a protein leading to resistance to sulfonamides (Rådström and Swedberg, 1988). The other two plasmids (from meconium and broilers) contained in the corresponding region of the plasmid section, additionally to the *sul2* gene, the resistance genes *dfrA17* and *aadA5* (trimethoprim and streptomycin/spectinomycin resistance; White et al., 2000) embedded in a partial class 1 integron devoid of the complete 3′-conserved structure.

A second antimicrobial resistance region identified in all three plasmids was the IS*Ecp1*-*bla*_CTX-M-1_ module. It is inversely inserted in the plasmid isolated from the meconium sample (pHV114.1; see **Figure 1**).

Another difference between pHV295.1, pHV114.1, and pHV292.1 is the insertion of an IS*66*-like element into pHV292.1 leading to the disruption of *umuC*, a gene of the UV protection operon (Perry et al., 1985).

## DISCUSSION

The hypothesis of a non-clonal dissemination of ESBL-encoding genes in Swiss poultry flocks is strengthened by the fact that ESBL-producing *E. coli* with different MLST ST and belonging to different phylogroups were isolated in the poultry production pyramid. Furthermore, the sequenced plasmids isolated from these *E. coli* were all highly similar. Moreover, sequences of three further IncI1 plasmids from ESBL-producing *E. coli* isolated in 2012 from broiler flocks in Switzerland (Wang et al., 2014) showed remarkable similarities with those reported here. All had the same backbone structures as well as the identical IS*Ecp1*-*bla*_CTX-M-1_ module and harbored also an incomplete class 1 integron containing a gene cassette composed of the trimethoprim and streptomycin/spectinomycin resistance genes.

When Leverstein-van Hall et al. (2011) investigated retail chicken meat and poultry samples, they identified a *bla*_CTX-M-1_ gene located mainly on IncI1 plasmids, but in contrast to our study, these plasmids belonged to the pMLST ST7. Similarly, in two Scandinavian studies vertical transmission of extended-spectrum cephalosporin resistant *Enterobacteriaceae*, mainly CMY-2-producing *E. coli* was reported (Agersø et al., 2014; Nilsson et al., 2014). These observed differences in the latter studies might be explainable by the different origin of the broiler breeder grandparents since the grandparents from the Swedish and Danish study originated from Sweden while those investigated in this study were from France. Dahmen et al. (2012) have confirmed earlier that IncI1/ST3 plasmids mainly contribute to the dissemination of *bla*_CTX-M-1_ in France. Furthermore, it has been shown, that IncI1 plasmids harboring *bla*_CTX-M-1_ show no or negligible fitness cost in *E. coli* and that these plasmids can persist in the absence of antimicrobial selection (Fischer et al., 2014). It is also known that plasmid-encoded elements are beneficial for stable inheritance of the plasmids, such as toxin/antitoxin systems or type IV pili which are beneficial to the bacteria for invasion and adhesion to the host gut (Carattoli, 2009).

Vertical transmission of ESBL-producing strains with these specific plasmids through the broiler production pyramid is only one possible explanation for their dissemination. Additionally, horizontal transmission of ESBL-producers and their plasmids within a poultry flock (coprophagic birds) plays another important role in their spread (Agersø et al., 2014; Nilsson et al., 2014). This factor might further explain the high diversity of different *E. coli* MLST types found in our study. The presence of *E. coli* of ST 1056 in all five sets, however, also highlights that some *E. coli* types might be more adapted to the poultry gut. This specific *E. coli* MLST has also been detected previously on Swiss retail poultry meat (Abgottspon et al., 2014). Moreover, we detected *E. coli* ST10 and ST117, two ST which are also considered poultry-associated (Leverstein-van Hall et al., 2011; Voets et al., 2013).

The final aspect to be considered focuses on the potential for co-selection via antimicrobial agents other than cephalosporins. Agersø et al. (2014) showed recently that to some extent extended-spectrum cephalosporinases-producing *E. coli* were selected by the use of aminopenicillins. The plasmids sequenced in this study harbored all of the *sul2* gene, which encodes resistance to sulfonamides. However, since in Switzerland at an average only 1 of 10 poultry flocks have to be treated with antibiotics and if so, fluoroquinolones and not β-lactam-antibiotics or sulfonamides are applied, the co-selection aspect may not really contribute to a further spread of these plasmids.

In conclusion, this study provides strong evidence that highly similar IncI1/ST3 plasmids harboring the *bla*_CTX-M-1_ gene are transmitted vertically in the broiler production pyramid from the top to the bottom with little evidence of any antimicrobial selection pressure. Intervention actions to reduce the risk of spreading ESBL-producing *E. coli* containing these plasmids have mainly to be focused on the top of the poultry production pyramid (nucleus poultry flock level). This situation should now be re-assessed in light of these findings.

## Conflict of Interest Statement

The authors declare that the research was conducted in the absence of any commercial or financial relationships that could be construed as a potential conflict of interest.

## Footnotes

1 http://www.lahey.org/studies/

2 http://www.ncbi.nlm.nih.gov/blast/

3 http://mlst.warwick.ac.uk/mlst/dbs/Ecoli
